# Additive Manufacturing of a Frost-Detection Resistive Sensor for Optimizing Demand Defrost in Refrigeration Systems

**DOI:** 10.3390/s24248193

**Published:** 2024-12-22

**Authors:** Martim Lima de Aguiar, Pedro Dinis Gaspar, Pedro Dinho da Silva

**Affiliations:** 1 Department of Electromechanical Engineering, University of Beira Interior, Rua Marquês d’Ávila e Bolama, 6201-001 Covilhã, Portugal; martim.aguiar@ubi.pt (M.L.d.A.); dinis@ubi.pt (P.D.G.); 2 C-MAST—Center for Mechanical and Aerospace Science and Technologies, 6201-001 Covilhã, Portugal

**Keywords:** additive manufacturing, resistive sensor, fused filament fabrication, frost detection, demand defrosting, heat exchanger, temperature sensor, 3D printing, refrigeration systems, conductive filament

## Abstract

This article presents the development of a resistive frost-detection sensor fabricated using Fused Filament Fabrication (FFF) with a conductive filament. This sensor was designed to enhance demand-defrost control in industrial refrigeration systems. Frost accumulation on evaporator surfaces blocks airflow and creates a thermal insulating barrier that reduces heat exchange efficiency, increasing energy consumption and operational costs. Traditional timed defrosting control methods can mitigate these effects but often lead to inefficiencies due to their inability to align with actual frost accumulation, which can vary according to system and environmental conditions. Frost-detection sensors aim to solve this problem by acting as a tool to support defrosting control. A series of tests were conducted to evaluate the sensor’s performance in detecting frost under controlled conditions on a heat exchanger (HX). The sensor reliably detected frost in all cases, demonstrating its effectiveness in real-time frost detection. The sensor measurements were validated by comparison with results obtained through a computer vision method, confirming its reliability. It was also found that the sensor can detect temperature changes. This advancement in sensor technology highlights the potential of additive manufacturing to provide cost-effective, customizable, replicable, and compact sensor designs, contributing to improved system performance and energy efficiency in refrigeration systems.

## 1. Introduction

Energy efficiency contributes to the reduction of operational costs and environmental impacts, particularly in energy-intensive processes such as refrigeration. The agri-food sector is responsible for a significant slice of the world’s energy consumption, which highlights the importance of optimizing energy usage and implementing sustainable practices. Studies have shown that processes such as refrigeration and cooking are among the largest consumers of electricity in food production, emphasizing the need for energy management strategies [1,2]. Enhanced monitoring, process optimization, and the adoption of renewable energy sources have been identified as essential measures to achieve energy savings and sustainability goals [3]. Moreover, disparities in energy consumption across industries underscore the potential for implementing equipment upgrades and process redesigns to improve efficiency [4]. Improving refrigeration systems by addressing inefficiencies, such as optimizing defrosting strategies, can enhance energy efficiency and reduce greenhouse gas emissions.

**Figure 1:**
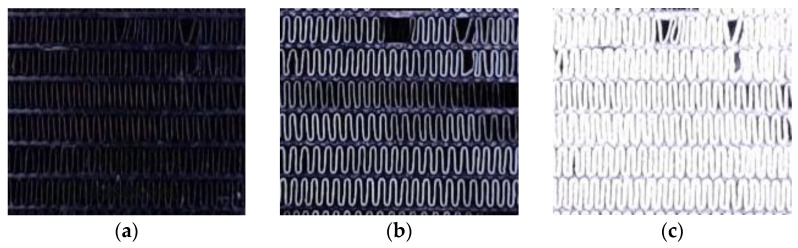
Stages of frost formation on the intake side of a heat exchanger (HX) showing minimal accumulation (**a**), initial frost formation (**b**), and frost saturation (HX blockage) (**c**).

Frost formation on heat exchanger (HX) surfaces, particularly on fin-and-tube evaporators, significantly impacts the efficiency of refrigeration systems by increasing thermal resistance and obstructing airflow, as shown in Figure 1. This phenomenon leads to a reduced heat transfer rate, heightened energy consumption, and operational inefficiencies [5,6]. Traditional defrosting methods, often based on preset time intervals, can either waste energy by initiating unnecessary defrost cycles or allow frost to accumulate excessively before activation, further compromising system performance [7].

The optimization of defrost cycles has been a focus of research for decades, to address the challenges posed by uneven frost deposition driven by factors such as surface temperature, air humidity, and flow characteristics. Over time, the frost layer’s changing thickness and density further degrade heat transfer and increase airflow resistance [8].

To mitigate these effects, periodic defrosting is employed, typically triggered by predetermined intervals or thresholds. However, traditional approaches often lack efficiency, either initiating defrost cycles prematurely or allowing excessive frost accumulation. Effective defrosting requires careful energy management, balancing the heat input necessary to melt frost against losses incurred during preheating of the cooler. This balance determines defrosting efficiency, which varies with frost density and the applied defrosting method.

Cooling cycle efficiency models integrate parameters such as operating time, cooling and defrost durations, and heat transfer rates to optimize defrost schedules. These models aim to calculate the ideal defrost initiation and duration, minimizing inefficiencies caused by under- or over-defrosting [8]. It has been found that there is an optimal runtime for defrost cycles that maximizes cooling efficiency. Factors such as fin spacing were also found to influence frost accumulation and system performance [9].

Intelligent control strategies have advanced defrost optimization. Demand-defrost strategies using neural network models to predict frost accumulation and determine optimal defrost initiation times have been shown to reduce unnecessary defrost events, improve temperature stability, and minimize energy consumption, demonstrating the practical feasibility of integrating advanced predictive control systems in retail refrigeration settings [10].

However, to address the complexities in the prediction of frost formation, demand defrosting strategies based on frost measurement rather than prediction have been proposed, aiming to optimize defrost cycles by aligning them with actual frost accumulation [11]. Various sensing technologies have been explored, including optical fiber, capacitive, piezoelectric, and resistive sensors [11,12].

Among these sensing technologies, capacitive sensors have demonstrated strong potential for frost detection. These sensors have shown the ability to detect frost, regardless of surface properties or temperature [13], and to capture real-time frost growth dynamics, growth stages, and the impact of varying operating conditions such as air temperature, velocity, and surface temperature in frost growth. Notably, capacitance readings revealed distinct shifts during the transition from condensation to solidification and crystal growth stages, supporting their potential for optimizing defrost control [14]. Capacitive sensors have also shown high accuracy in mapping frost distribution over various fin structures. However, to achieve this precision, calibration and layout optimization are required for precise frost measurements and efficient defrost control [15].

Resistive sensors offer a combination of simplicity, robustness, cost-effectiveness, and adaptability. These sensors operate by measuring the electrical resistivity of the medium between electrodes, leveraging the significant resistivity differences among air, water, ice, and frost [16]. While effective in principle, their performance depends heavily on design considerations such as electrode material, spacing, and placement within the heat exchanger [17].

The rise of additive manufacturing technologies, such as Fused Filament Fabrication (FFF), has provided novel and accessible tools for developing cost-effective and advanced sensors. FFF enables the fabrication of customizable, compact, and cost-effective sensors by utilizing conductive composite filaments. This approach allows the design of customized sensors, facilitating integration into complex systems such as heat exchangers without significantly disrupting airflow. Recent studies have demonstrated the feasibility of using FFF to create various sensor types, including piezoresistive and capacitive displacement sensors and electrically conductive circuits, showcasing their potential for low-cost, embedded, and easily replicable applications [18,19,20,21,22,23]. For instance, Stefanov et al. (2022) fabricated a capacitive sensor with graphene/PLA composites, achieving high sensitivity and flexibility [18], while Masarra et al. (2022) demonstrated enhanced conductivity in FFF-produced PLA/PCL/graphene nanoplatelet composites compared to injection-molded alternatives [19], and Ragolia et al. (2021) demonstrated a strong correlation between resistance and temperature changes in commercial FFF conductive filaments, highlighting the sensitivity of 3D printed resistive sensors to thermal variations [20]. These advancements mark the rise of an era in which additive manufacturing allows the production of accessible, customized sensors customized to diverse operational requirements, provided the working principles are thoroughly understood during the design phase.

Previous works have also explored resistive sensors for frost detection, highlighting their simplicity and adaptability in refrigeration systems. These sensors operate by measuring the resistance between electrodes, based on the principle that distinct resistivity differences among air, water, ice, and frost allow for the detection of the material between electrodes [11,17,24]. Studies have identified key design considerations, including electrode material, spacing, and sensor placement within the HX, to ensure accurate detection under varying environmental conditions. While resistive sensors have shown promise in frost-detection applications, challenges remain in optimizing their design for real-world implementations, particularly regarding their integration with complex geometries and systems. This study builds on these foundations using the understanding of the principles that allow for frost detection using resistive sensors, together with the potential of additive manufacturing to enhance sensor design, fabrication, and placement flexibility, aiming to address the limitations of traditional resistive sensor manufacturing technologies, to enhance frost-detection technologies.

Using thermoplastic polyurethane (TPU) composite conductive filament, sensors can be fabricated using FFF and designed to fit the required geometries and installed in tight spaces between HX fins within heat exchangers. This approach minimizes disruption to airflow while enhancing detection accuracy.

In this study, a resistive frost-detection sensor fabricated via FFF additive manufacturing is presented and evaluated. Laboratory tests were conducted to assess its performance in detecting frost under controlled conditions. The sensor measurements were compared against results obtained using a computer vision-based method previously developed [24], providing a baseline for accuracy and reliability. The findings highlight the potential of additive manufacturing for advancing frost-detection technologies, contributing to more efficient and sustainable refrigeration systems.

This research addresses the limitations of traditional defrosting methods and highlights the growing role of additive manufacturing in developing innovative sensor solutions. By measuring frost and defrosting this work allows for the adaptation of the frequency and duration of defrost cycles to the actual defrosting needs, offering a pathway to enhancing energy efficiency and operational reliability in refrigeration systems.

## 2. Materials and Methods

This section outlines the principles, design, and fabrication of a resistive frost-detection sensor developed using additive manufacturing techniques. The sensor was tested in controlled conditions on a HX and validated against a computer vision method. Details on the sensor’s working principles, 3D printing process, electrical measurement setup, and validation methodology are provided.

### 2.1. Experimental Setup

**Figure 2:**
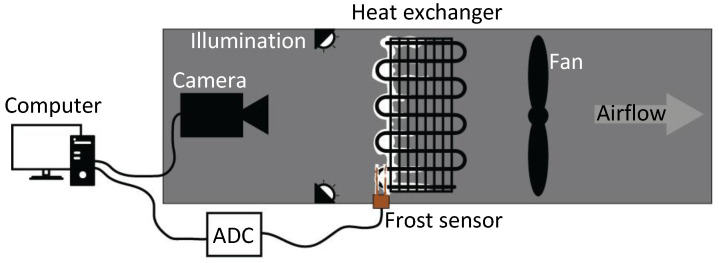
Schematic representation of the experimental setup.

The experimental setup involved placing the resistive frost-detection sensor at the intake side of the heat exchanger (HX), where frost formation is larger. The sensor was connected to an Analog-to-Digital Converter (ADC), which interfaced with a PC for data acquisition and analysis. A camera was positioned to face the HX intake, capturing images of its surface to monitor frost accumulation and validate sensor readings. Illumination was directed toward the HX to reduce external interference and enhance image quality, while a fan circulated air through the HX to replicate real-world operational conditions. Prior to recirculation through the HX, the air was humidified by passing it through a passive humidifier, which was recharged before the start of each test. The layout of the sensor, camera, and HX intake is depicted in Figure 2.

Both the camera and the ADC were connected to a PC running MATLAB, which processed the images and stored the collected data for further analysis.

### 2.2. Working Principles of a Frost-Detection Resistive Sensor

**Figure 3:**
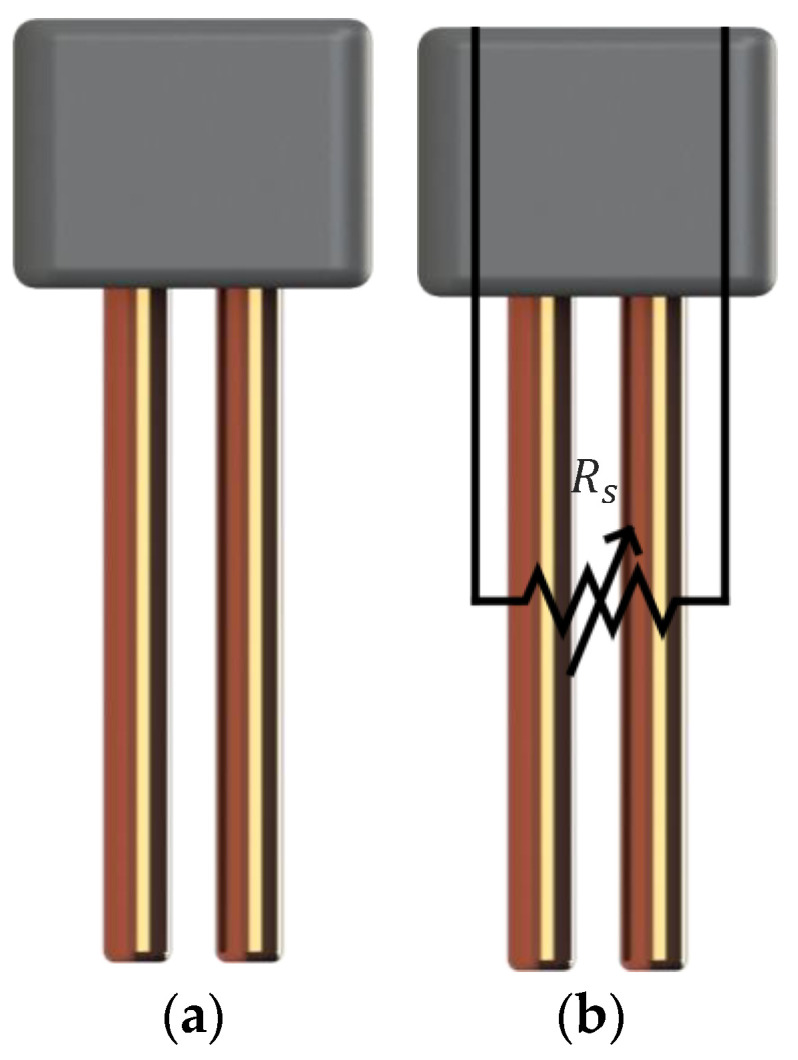
Simplified representation of the resistive sensor (**a**) with overlaid equivalent circuit representation (**b**).

The resistive frost-detection sensor operates based on the principle of varying electrical resistance between two electrodes. In previous studies, two copper electrodes were used in a configuration whose working principle is shown in Figure 3.

**Figure 4:**
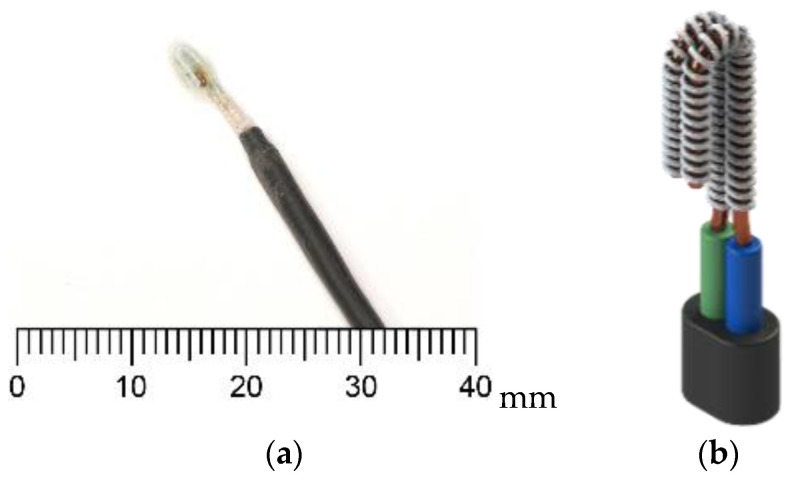
Resistive frost-detection sensor (**a**) and CAD model (**b**), as described in [17].

The resistivity of the electrode medium changes according to the presence of different materials—air, water, or frost. When condensation or frost forms between the electrodes, the electrical resistivity changes significantly due to the distinct resistivity values of these materials. By measuring overall sensor resistance, it is possible to infer the presence of condensation or frost accumulation on the heat exchanger (HX) surface. In previous studies, a copper wire electrode sensor with a fabric medium between the electrodes demonstrated the best frost-detection performance among all resistive sensor mediums tested. This sensor is depicted in Figure 4.

The objective of this work was to develop a 3D-printed resistive sensor capable of detecting frost on HX fins, leveraging the advantages of additive manufacturing technology. The sensor was fabricated using a conductive filament that combines electrical conductivity with flexibility, enabling the production of a device that can be wrapped around the fins. This approach enables the accessible fabrication and easy replicability of a precise, miniaturized, and flexible sensing device with high compatibility with the complex geometries of HX while minimizing interference with airflow.

### 2.3. Acquisition Circuit

**Figure 5:**
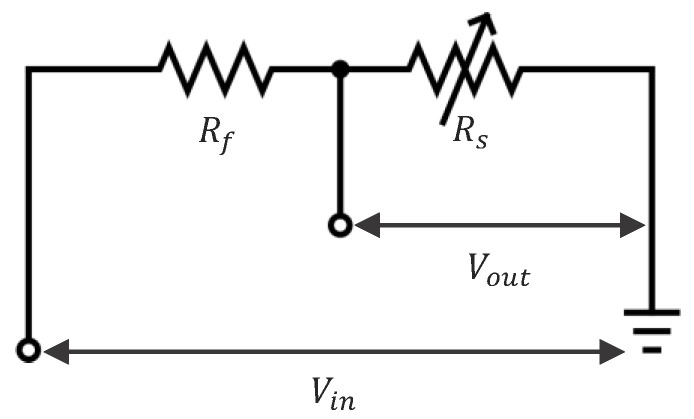
Voltage divider circuit for sensor resistance measurement.

To measure the variable resistance of the sensor ($R_{s}$), a voltage divider circuit was implemented, as represented in Figure 5.

The circuit consists of a fixed resistor ($R_{f}$), in series with the sensor ($R_{s}$), connected between a voltage supply ($V_{in}$), and ground. The output voltage ($V_{out}$), across the sensor is given by Equation (1):

(1) $$V_{out}=V_{in}\frac{R_{s}}{R_{s}+R_{f}}$$

An Arduino microcontroller with a 10-bit ADC was used to convert analog voltage signals into digital values, represented as a dimensionless unit ranging from 0 to 1023, corresponding to voltages between 0 V to 5 V ($V_{in}$). By rearranging the voltage divider equation, the sensor resistance can be calculated:

(2) $$R_{s}=\frac{V_{out}\times R_{f}}{V_{in}-V_{out}}$$

Despite this, direct ADC values were plotted instead of calculating resistivity, as when $V_{in}-V_{out}$ approaches zero, $R_{s}\;$tends to infinity. Given that air has an extremely high resistance, this approach allows for the representation of both very high resistivity values for air and smaller resistance fluctuations for water within the same plot, providing a more comprehensive understanding of the sensor’s behavior across different mediums. Furthermore, in this configuration, $R_{s}$ increases with the increase in the sensor resistivity. At higher resistance values, the precision of the voltage divider circuit decreases due to the nature of the voltage divider and the ADC’s limited resolution. This limitation contributes to the observed fluctuations in resistance readings. To mitigate the effect on results, resistance values used for calculations were averaged over 10 readings to reduce the impact of measurement noise and enhance accuracy.

### 2.4. Temperature Coefficient of Resistance

The Temperature Coefficient of Resistance (TCR) quantifies how the electrical resistance of a material changes with temperature. It is defined by Equation (3):

(3) $$TCR=\frac{\Delta R}{R_{0}\Delta T}$$

where Δ*R* is the change in resistance, $R_{0}$ is the resistance at the reference temperature $T_{0}$, and ΔT is the change in temperature. Understanding the TCR is helpful in the context of the acquisition circuit, as it allows for the assessment of temperature-induced variations in the sensor’s resistance. Since the resistance of the conductive polymer composite used in the sensor changes with temperature [20], calculating the TCR enables the understanding of the necessity of compensation strategies. This ensures that resistance changes attributed to frost accumulation are not confounded by temperature variations, enhancing the accuracy of the voltage measurements obtained from the voltage divider circuit and improving the reliability of frost detection in the refrigeration system.

### 2.5. Design of a Resistive Frost-Detection Sensor for Additive Manufacturing

**Figure 6:**
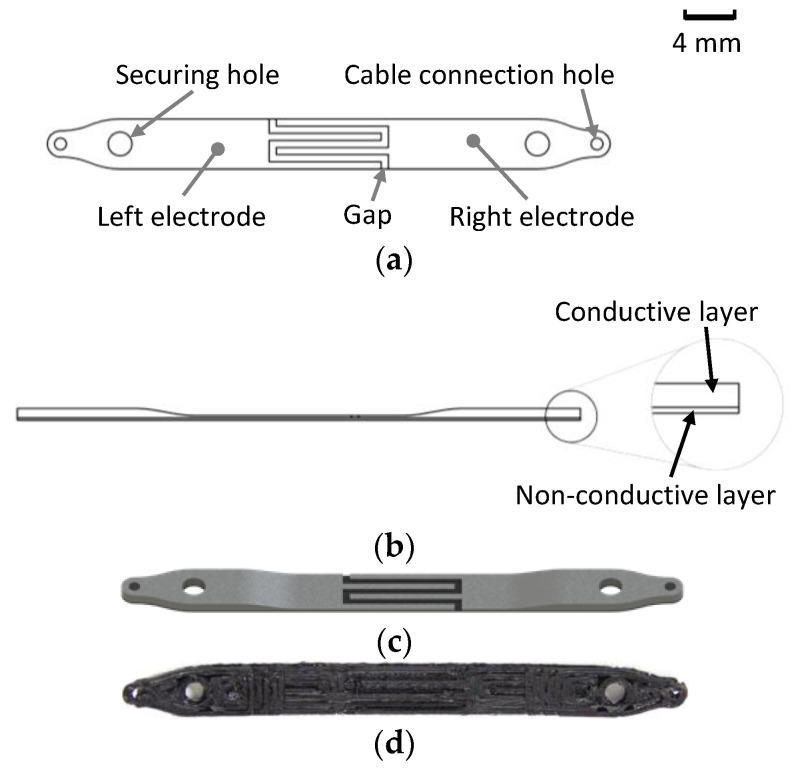
Front view (**a**), side view (**b**), rendered view (**c**), and photograph (**d**) of the 3D-printed resistive frost-detection sensor.

The sensor consists of three main components: an electrically insulating Filaflex TPU95A base layer, upon which two conductive circuits are printed using Filaflex conductive TPU92A, as depicted in Figure 6a. The circuits are arranged in an interdigitated electrode pattern. This design enhances the shared surface area between the electrodes, enhancing sensitivity by increasing the region where condensation, frost, or defrosting water can bridge the small gap between the circuits.

The use of Fused Filament Fabrication (FFF) technology for sensor fabrication introduces limitations based on the nozzle diameter, which in this case was 0.6 mm, though this parameter can be adjusted by changing the nozzle. The sensor was designed to be optimized for manufacturing characteristics (design for additive manufacturing—DFAM), including a layer height of 0.2 mm and a nozzle diameter of 0.6 mm. As a result, the insulating base layer is 0.2 mm thick, while the conductive circuit layer also has a thickness of 0.2 mm and 0.6 mm width. The gap between the electrodes can be customized independently of the nozzle diameter. For this study, a gap of 0.5 mm was selected. At the sensor’s extremities, the conductive layer is thicker, reaching 0.8 mm. This increased thickness enhances mechanical durability in the fixture regions and improves electrical contact between the sensor electrodes and the connecting cables. This profile is illustrated in Figure 6b.

**Figure 7:**
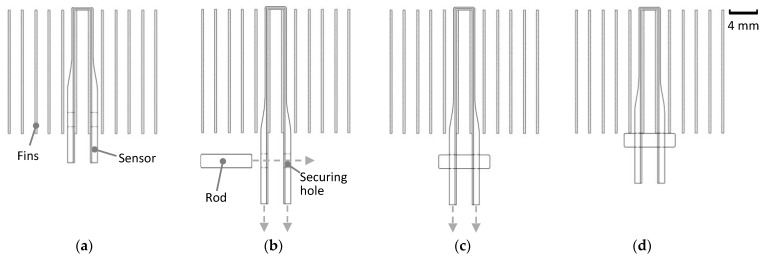
Sensor mounting process: placement of the sensor between HX fins (**a**), stretching of the sensor (**b**), insertion of the mounting rod for attachment (**c**), and mounted sensor (**d**).

To install the sensor, it includes two holes at each extremity: one larger and one smaller. The larger hole is designed for securing the sensor to HX fins by wrapping it around the fin and passing a non-conductive rod through both holes to fix it in place. The mounting procedure is detailed in Figure 7

**Figure 8:**
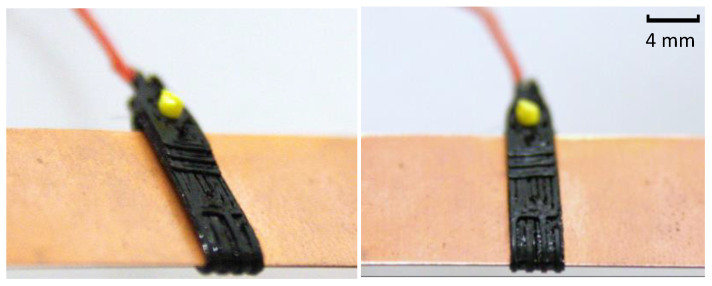
Sensor fixed to a copper fin.

First, the sensor is placed between the HX fins, as shown in Figure 7a. Due to the flexibility of TPU, the sensor can be stretched by pulling its extremities, as illustrated in Figure 7b. Next, a non-conductive rod is inserted into the securing hole, as shown in Figure 7c, and the pulling force is released, allowing the sensor to remain stretched and securely mounted against the fins, as shown in Figure 7d. This flexibility allows stretching and minimizes the air gaps between the sensor and the fins. Reducing these air gaps enhances thermal coupling, as even microscale air gaps can act as insulating barriers, lowering the effectiveness of heat transfer. A closer physical contact means the sensor is better able to respond thermally to changes in the HX surface, improving its ability to detect frost formation and condensation at rates comparable to the HX fins. The sensor mounted in a single copper fin is shown in Figure 8.

An Artillery Sidewinder X1 3D printer was used for fabricating the sensor. This printer is recommended by the filament manufacturer for printing flexible materials as it is equipped with a direct drive extruder. The sensor fabrication involved a dual-material printing process, in which the first layer was printed using non-electrically conductive Filaflex TPU 95A, the print was paused, the filament was changed to the conductive filament, and then the print was resumed.

The total thickness of the sensor in the sensing region was 0.4 mm, making it thin and flexible. This design allows the sensor to adjust to various HX geometries and be easily attached around the fins without significantly obstructing airflow.

Among the main FFF materials, TPU stands out with the highest thermal conductivity of 0.26 ± 0.05 W/m·K [25], making it a relatively poor thermal insulator. This characteristic, together with the thinness of the sensor, reduces its overall thermal resistance. Low thermal resistance allows the sensor to transfer sufficient heat with the HX for water condensation and frost formation to happen on the sensor at approximately the same rate as on the HX surface.

### 2.6. Computer Vision Method for Validation

**Figure 9:**
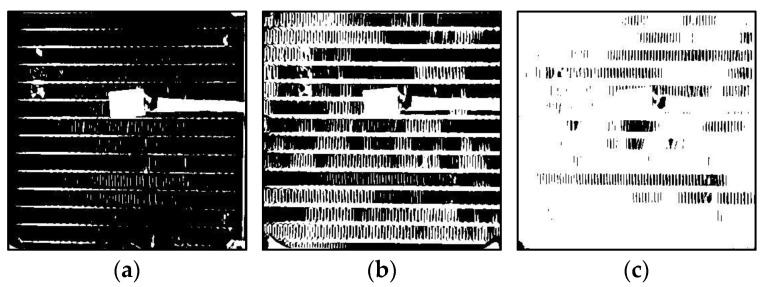
Stages of frost formation on the HX captured by the computer vision method: no frost detected (**a**), partial frost accumulation (**b**), and almost complete frost coverage (**c**).

To validate the sensor’s performance, measurements were compared with data obtained from a previously developed computer vision (CV) method [24]. This method involves capturing images of the HX surface, cropping them to the HX intake surface, and processing them to detect frost accumulation. This method processes images with a resolution of 351 × 351 pixels, where each pixel is classified as either “frost” or “no frost” based on a predefined threshold that accounts for the HX color and lighting conditions. The total number of pixels identified as frost is then normalized to a scale from 0 to 1023, ensuring compatibility with the ADC’s resolution and facilitating direct comparison with the sensor’s measurements, as shown in Figure 9.

The CV method easily detects frost (white) but has difficulty detecting water (transparent), which makes it almost impossible to detect condensation and water from melting frost. A dry HX indicates the requirement to stop a defrosting cycle. Thus, the capability of the resistive sensor to detect water is an advantage.

### 2.7. Experimental Procedures

To evaluate the sensor’s performance, experiments were designed to assess its capability to measure frost accumulation and removal, focusing on its responsiveness and reliability under controlled conditions.

Frost Formation: To initiate frost formation, the refrigeration system was turned on, and the fan was set to operate at a low speed to encourage frost accumulation on the heat exchanger (HX) intake. The system was allowed to run continuously while the camera monitored the frost accumulation. Frost formation was tracked using the computer vision method, and once the frost-detection value reached 950, a defrosting cycle was triggered.

Defrosting: During the defrosting cycle, the refrigeration system was turned off, and the fan speed increased to its maximum setting to facilitate the removal of frost. This process was maintained for 15 min, after which the system returned to its initial state to prepare for subsequent experiments.

With this setup, data were collected and analyzed, comparing the results from the resistive sensor to those obtained from the computer vision method.

## 3. Results

**Figure 10:**
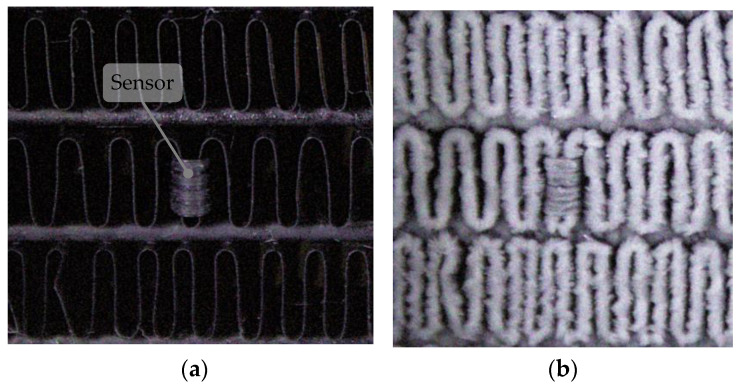
Images of the sensor placed in the HX with dry HX with no frost formation (**a**) and with frost accumulation on both the HX and the sensor surfaces (**b**).

The sensor was positioned in the bottom left corner of the HX, near the working fluid outlet, and the refrigeration system was activated. While frost formation across the entire HX intake surface is not uniform due to varying conditions across it, frost accumulation within small, localized regions, including the area around the sensor, displayed a consistent pattern. As shown in Figure 10, frost accumulates on the sensor surface similarly to the accumulation in the nearby HX fins, suggesting the sensor can represent the regional frost conditions. This alignment contributes to the effectiveness of the sensor as a representative tool for detecting frost formation in its immediate surroundings.

In this section, the performance of the resistive frost-detection sensor is evaluated through four frost-defrost cycles, referred to as cycles 1, 2, 3, and 4. The sensor readings and CV method outputs were recorded over time and plotted for each cycle. The resulting plots illustrate the ADC values obtained from the sensor and the CV method, with the x-axis representing time in minutes and the y-axis representing the ADC values. The background of each plot is shaded gray during defrosting operations and left white during frost formation periods.

### 3.1. Cycle 1

**Figure 11:**
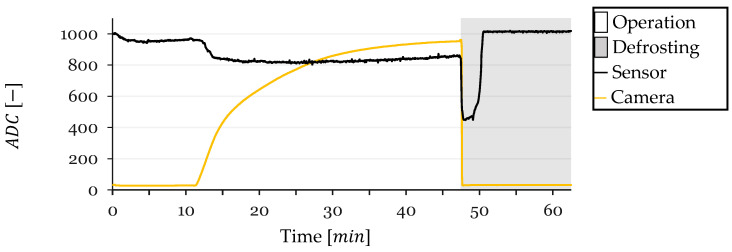
Sensor and CV Method ADC Values over Time for Cycle 1.

At the onset of Cycle 1, the sensor’s ADC value decreased from approximately 1010 to 950 due to initial condensation on the sensor surface (Figure 11). This value stabilized around 950, with a slight continuous increase likely attributed to a gradual temperature decrease affecting the sensor’s resistance. At t=12 min, a sharp drop in the sensor’s ADC value was observed, indicating the onset of frost formation between the electrodes. Concurrently, the CV method began detecting frost, confirming the sensor’s response to actual frost accumulation.

The sensor readings saturated at t=14 min, reaching a minimum ADC value, while the CV method recorded a frost-detection value of 331, which continued to increase sharply. Following saturation, the sensor readings exhibited a very slow decrease, reaching a minimum ADC value of 820 at t=30 min, when the CV method indicated a frost-detection value of 875.

Upon the CV method reaching a frost-detection value of 950 at t=47 min, a defrosting cycle was initiated. During defrosting, the CV value dropped close to zero, and the sensor’s ADC value experienced a sharp decrease from 860 to 448 within a few seconds, corresponding to the melting of frost and the presence of liquid water bridging the electrodes. The sensor’s ADC value remained low while water was present and returned to the initial value of approximately 1010 after 3.5 min of defrosting once the HX surface dried. The sensor maintained this value until the end of the test.

**Figure 12:**
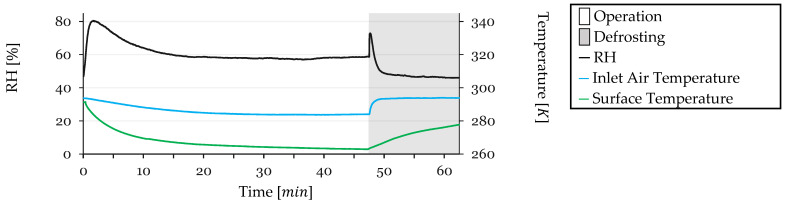
Monitored test conditions for Cycle 1: inlet air RH and temperature and surface temperature.

Some test conditions were monitored during testing. Inlet air temperature, refrigerant outlet temperature (closely approximating the HX surface temperature due to proximity to the sensor), and inlet air Relative Humidity (RH) were monitored. These parameters are shown in Figure 12 for Cycle 1.

The RH exhibits a sharp spike at the beginning of the test due to the initial recirculation of humidified air. It reaches a maximum of 80%, then begins to decrease as the HX surface temperature drops to the dew point, initiating condensation on the HX surface and dehumidifying the air. After 20 min, a balance is achieved between the humidifier’s capacity to humidify the air and the HX’s capacity to remove moisture, stabilizing at approximately 58%. During defrosting, when the system is open to the external air, warmer intake air enters the circuit, leading to an increase in RH to 78%, which quickly drops back, reaching 62% as the HX dries and the system stabilizes, by the end of the cycle.

Inlet air temperature starts at 293 K and gradually decreases throughout the test, stabilizing at 284 K. During defrosting, as warmer air enters the circuit, the temperature rises, stabilizing at 294 K after 2 min of defrosting.

The HX surface temperature, measured as the refrigerant outlet temperature, starts at 294 K and initially decreases rapidly as the refrigeration system is reactivated post-defrosting. It reaches 266 K by 20 min, continuing a slower decline as frost accumulation reduces heat transfer efficiency, stabilizing just before defrosting. During defrosting, the temperature rises to 278 K. While discrepancies between surface temperature and refrigerant temperature are possible during defrosting due to halted refrigerant flow, the high flow rate during operation ensures equivalence between measured and actual surface temperatures during refrigeration phases.

### 3.2. Cycle 2

**Figure 13:**
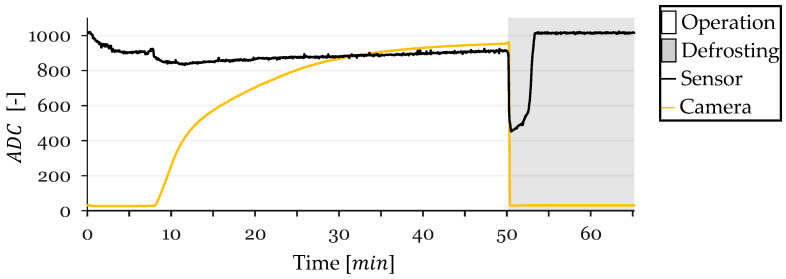
Sensor and CV Method ADC Values over Time for Cycle 2.

In Cycle 2, the sensor’s ADC value decreased from 1010 to around 900 due to condensation, stabilizing at this level with a slight increase over time (Figure 13). At t=8 min, the sensor exhibited a sharp drop in ADC value, indicating frost formation, coinciding with the CV method’s initial frost detection. The sensor readings saturated at t=12 min with a corresponding CV frost-detection value of 394.

Post-saturation, the sensor readings showed a very slight increase, likely due to continued temperature decrease affecting resistance. Upon the CV method reaching a frost-detection value of 950 at t=50 min, defrosting commenced. The sensor’s ADC value dropped sharply from 907 to 467, remaining low but gradually increasing as water was present. After approximately 3 min, the sensor’s ADC value returned to the initial level of around 1010 once the HX dried, maintaining this value until the test concluded.

**Figure 14:**
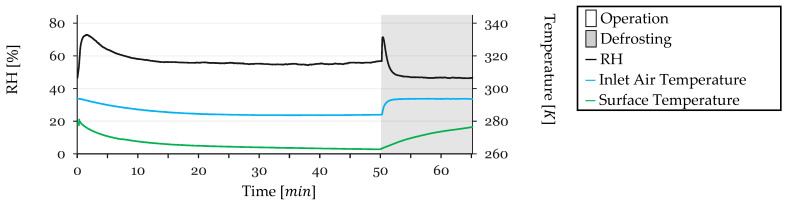
Monitored test conditions for Cycle 2: inlet air RH and temperature and surface temperature.

Test conditions, with inlet air temperature, refrigerant outlet temperature, and inlet air RH, were monitored throughout the cycle. The recorded data are presented in Figure 14 for Cycle 2.

RH peaks at 73% at the start and stabilizes at 56% by 15 min as condensation begins to dehumidify the air. During defrosting, RH peaks again at 70%, then decreases to 47% as the HX dries.

Inlet air temperature begins at 294 K and decreases gradually, stabilizing at 285 K. During defrosting, the temperature increases to 294 K, stabilizing at this level after 2 min.

HX surface temperature starts at 279 K and decreases steadily, reaching 266 K after 15 min. It continues to decline to 263 K before defrosting. During defrosting, the temperature rises to 278 K.

### 3.3. Cycle 3

**Figure 15:**
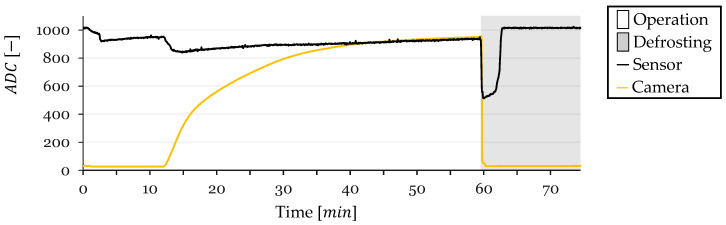
Sensor and CV Method ADC Values over Time for Cycle 3.

During Cycle 3, the sensor’s ADC value decreased from 1010 to approximately 920 due to condensation, stabilizing with a slight increase over time (Figure 15). A sharp drop in ADC value occurred at t=12.25 min, indicating frost formation, which aligned with the CV method’s frost-detection initiation. The sensor readings saturated at t=14.7 min while the CV method recorded a frost-detection value of 204.

Following saturation, the sensor readings exhibited a slight increase, again attributed to temperature-induced resistance changes. Defrosting was initiated when the CV method reached a frost-detection value of 950 at t=60 min. The sensor’s ADC value decreased sharply from 925 to 521, remaining low during the presence of water and returning to approximately 1010 after 3 min once the HX dried.

**Figure 16:**
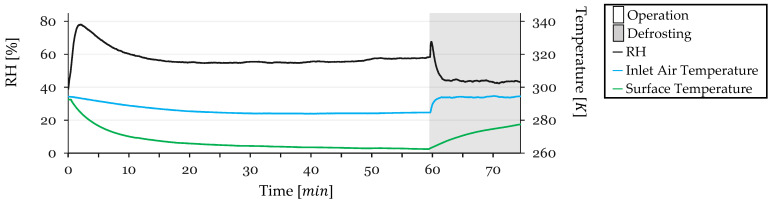
Monitored test conditions for Cycle 3: inlet air RH and temperature and surface temperature.

During Cycle 3, inlet air temperature, refrigerant outlet temperature, and inlet air RH were measured. These monitored parameters are depicted in Figure 16.

The RH starts with a peak of 78%, stabilizing at 55% after 20 min. Between minute 40 and the defrosting phase, RH slightly increases to 58%. During defrosting, RH peaks at 66%, decreasing to 43% after the HX dries.

Inlet air temperature starts at 294 K, gradually stabilizing at 285 K. It increases to 294 K during defrosting, stabilizing after 2 min.

The HX surface temperature starts at 294 K, decreases to 265 K after 20 min, and continues declining to 263 K before defrosting. During defrosting, the temperature rises to 277 K.

### 3.4. Cycle 4

**Figure 17:**
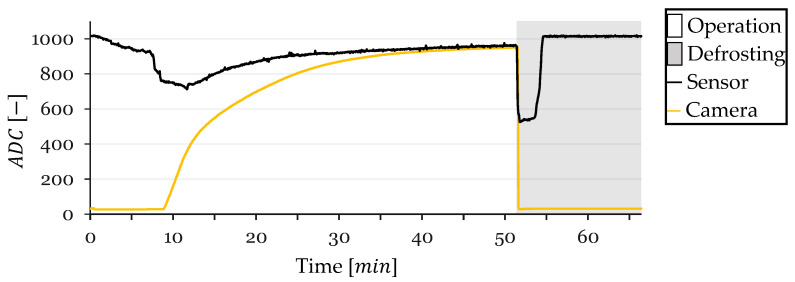
Sensor and CV Method ADC Values over Time for Cycle 4.

In Cycle 4, the sensor’s ADC value decreased from 1010 to about 925 due to condensation without significant stabilization (Figure 17). At t=7.2 min, the sensor experienced a sharp ADC drop, indicating frost formation occurring approximately one minute before the CV method detected frost. The sensor readings saturated at t=11.6 min with a CV frost-detection value of 310.

Post-saturation, the sensor readings increased slightly, likely due to temperature effects. Defrosting began when the CV method reached a frost-detection value of 950 at t=51.6 min. The sensor’s ADC value decreased sharply from 965 to 535, remaining low during water presence and returning to the initial value of approximately 1010 after 3.5 min once the HX dried.

The slight increases in sensor measurement values after saturation are likely influenced by temperature variations affecting the resistance of the conductive TPU material, as suggested by [20]. To assess this, a comparison was made between the temperature of the working fluid inside the HX and the sensor’s ADC values after frost-detection saturation.

**Figure 18:**
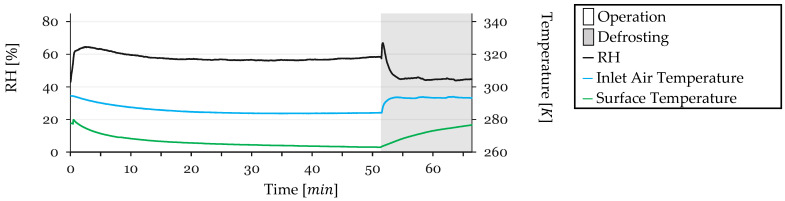
Monitored test conditions for Cycle 4: inlet air RH and temperature and surface temperature.

Inlet air temperature, refrigerant outlet temperature, and inlet air RH were monitored during Cycle 4. The data are summarized in Figure 18.

RH peaks at 65% initially, stabilizing at 57% by 20 min. After minute 30, RH slightly increases to 58% before defrosting. During defrosting, RH peaks at 66%, decreasing to 45% once the HX dries.

Inlet air temperature begins at 294 K and stabilizes at 285 K. During defrosting, it increases to 293 K and stabilizes after 2 min.

HX surface temperature starts at 278 K, decreases rapidly to 265 K after 20 min, and continues to decline to 263 K before defrosting. During defrosting, the temperature rises to 277 K.

### 3.5. Temperature Effect on Sensor Readings

An analysis was conducted to explore the effect of temperature on the sensor’s resistance readings during the steady-state region before defrosting. This period is characterized by lower changes in factors such as sensor humidity and sensor surface airflow, as the sensor is insulated by a stable frost layer. By examining this region, the objective is to determine if temperature variations significantly affect the sensor’s resistance and to assess the linearity of this relationship. Sensor resistance values were calculated from the ADC readings using Equation (2).

Due to the decreased precision at higher resistance values inherent in the voltage divider equation, the higher resistance data exhibited fluctuations. To mitigate this and enhance the clarity of trends, a moving average with a window of 20 values was overlaid with the resistance data. Temperature readings were obtained from a sensor measuring the temperature of the HX working fluid.

For each frost-defrost cycle, the last 10 min before defrosting were analyzed. This timeframe was chosen because the sensor’s resistance is primarily influenced by temperature variations during this steady-state period. Resistance (in MΩ) is plotted on the left y-axis, and temperature (in Kelvin) is plotted on the right y-axis, with the temperature axis presented in reverse order. This orientation aligns the direction of temperature decrease with the observed increase in resistance, facilitating a direct comparison of the slopes.

#### 3.5.1. Cycle 1—Resistance and Temperature

**Figure 19:**
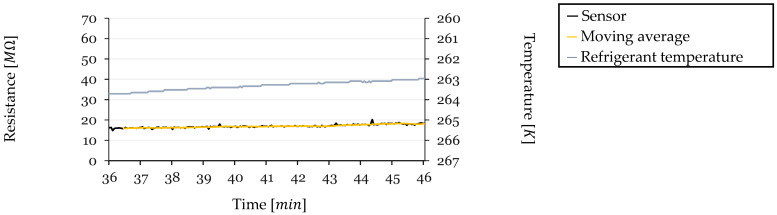
Resistance and Temperature vs. Time for Cycle 1 (Steady-State Region).

In Cycle 1 (Figure 19), the initial temperature at the start of the sample period was 263.71 K, and over the 10-min timeframe from 36 to 46 min into the cycle, the temperature decreased by ΔT=−0.71 K, reaching 263.00 K just before defrosting. Correspondingly, the sensor’s resistance increased from an initial value of $R_{0}=18.20\;M\Omega$ (calculated at 263.02 K) by ΔR=2.23 MΩ, culminating in a final resistance of approximately 20.43 MΩ. The TCR for this cycle was calculated using Equation (3) to be $-0.171\;K^{-1}$.

This TCR indicates a modest sensitivity of the sensor’s resistance to temperature changes during this cycle. Notably, Cycle 1 exhibited the weakest correlation between temperature decrease and resistance increase among all cycles analyzed. The slope of the resistance curve was less pronounced compared to that of the temperature curve. This discrepancy suggests that additional factors may have influenced the sensor readings, potentially including a slower saturation of frost detection or slight environmental variations that counteracted the expected temperature effect on resistivity. However, the overall effect was minimal, and a general trend of resistance increasing with decreasing temperature was still observable.

#### 3.5.2. Cycle 2—Resistance and Temperature

**Figure 20:**
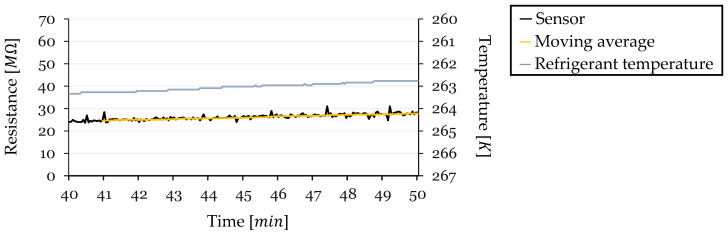
Resistance and Temperature vs. Time for Cycle 2 (Steady-State Region).

For Cycle 2 (Figure 20), the initial temperature was 263.32 K at the start of the sample period, which spanned from 40 to 50 min into the cycle. The temperature decreased by ΔT=−0.55 over this period. The sensor’s resistance began at $R_{0}=25.88\;M\Omega$(at 263.02 K) and increased by ΔR=3.50 MΩ, reaching approximately 29.38 MΩ just before defrosting. The calculated TCR for this cycle was $-0.246\;K^{-1}.$

In this cycle, a clearer correlation between temperature decreases and resistance increase was evident. The resistance values, despite exhibiting high-frequency but low-amplitude fluctuations due to the limitations of the voltage divider at high resistance levels, showed a consistent upward trend when smoothed with a moving average. This trend closely mirrored the temperature decrease, indicating that temperature variations had a more pronounced impact on the sensor’s resistance during this cycle compared to Cycle 1.

#### 3.5.3. Cycle 3—Resistance and Temperature

**Figure 21:**
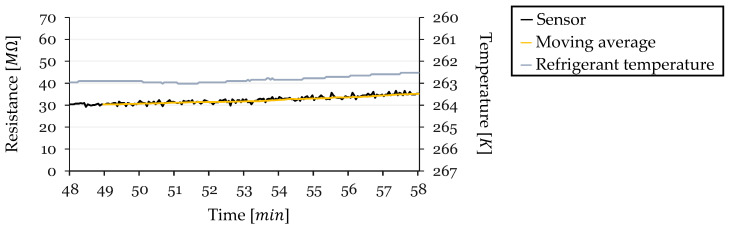
Resistance and Temperature vs. Time for Cycle 3 (Steady-State Region).

During Cycle 3 (Figure 21), the sample period from 48 to 58 min into the cycle began with an initial temperature of 262.93 K. The temperature decreased by Δ T=−0.41 over this 10-min interval. The sensor’s initial resistance was $R_{0}=31.42\;M\Omega$ (at 263.02 K), and it increased by ΔR=4.69 MΩ, reaching approximately 36.11 MΩ before defrosting. The TCR calculated for this cycle was $-0.364\;K^{-1}$, the highest among all cycles analyzed.

Cycle 3 exhibited a strong correlation between temperature and resistance. The resistance curve closely followed the temperature curve (in reverse order on the plot), and even minor fluctuations in temperature were reflected in the resistance readings. The high TCR indicates a significant sensitivity of the sensor’s resistance to temperature changes during this period. The moving average effectively highlighted the overall increasing trend in resistance corresponding to the decreasing temperature, suggesting that temperature was the dominant factor influencing resistance in this steady-state region.

#### 3.5.4. Cycle 4—Resistance and Temperature

**Figure 22:**
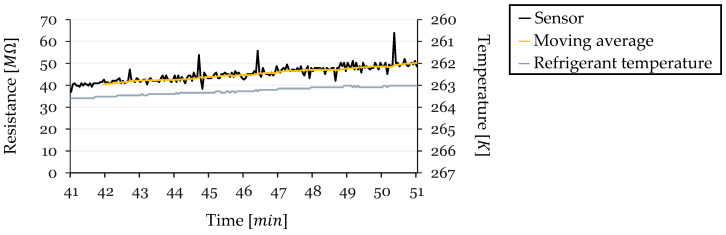
Resistance and Temperature vs. Time for Cycle 4 (Steady-State Region).

In Cycle 4 (Figure 22), the sample period extended from 41 to 51 min into the cycle, with an initial temperature of 263.59 K. Over this period, the temperature decreased by ΔT=−0.57 K. The sensor’s resistance started at $R_{0}=48.84\;M\Omega$ (at 263.02 K) and experienced the largest increase among all cycles, with ΔR=9.90 MΩ, reaching approximately 58.74 MΩ before defrosting. The TCR for this cycle was $-0.356\;K^{-1}$.

Cycle 4 displayed the most substantial fluctuations in resistance readings, with spikes reaching up to 15 MΩ. These fluctuations were more pronounced due to the sensor operating near the upper limits of the ADC’s resolution, where a small voltage divider $V_{out}$ changes lead to significant calculated resistance variations. Despite these fluctuations, the moving average revealed a noticeable upward trend, and the general correlation between decreasing temperature and increasing resistance was still apparent.

#### 3.5.5. Whole Cycle Resistance vs. Temperature Comparison

**Figure 23:**
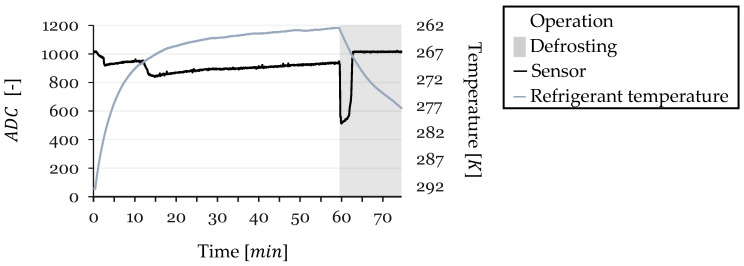
Sensor Resistance and Temperature vs. Time for Cycle 3 (Full Duration).

To further investigate the temperature influencing the sensor’s resistance beyond the steady-state region, an analysis was conducted for the entire duration of Cycle 3. Figure 23 presents the sensor’s ADC values alongside the temperature of the working fluid inside the HX throughout the cycle.

An analysis of the ADC values and temperature data reveals that a correlation between temperature and ADC readings is observable only during the steady-state region before defrosting. During phases of condensation, frost formation, and defrosting, no apparent relationship exists between these two variables. This indicates that while temperature variations are expected to always influence sensor readings, their influence is minimal compared to other factors that have a more significant effect. Consequently, they are not the predominant factor affecting ADC fluctuations during periods of rapid environmental changes. In these non-steady-state phases, factors such as the physical presence of condensation, frost accumulation, and the melting process during defrosting have a more significant impact on the sensor’s readings than temperature alone.

## 4. Discussion

The resistive frost-detection sensor developed in this study exhibited consistent responsiveness to condensation, frost formation, and defrosting across all four experimental cycles. During the condensation phase, an initial decrease in ADC values from approximately 1010 to a lower value was observed. This change is attributed to the formation of a conductive layer of condensed water bridging the sensor electrodes. Slight increases in ADC values over time during this phase were possibly due to temperature decreases affecting the resistance of the conductive polymer composite material. The sensor’s sensitivity to condensation highlights its capability to monitor the early stages of moisture accumulation, which is helpful for frost detection.

As frost formation commenced, the sensor demonstrated sharp drops in ADC values, coinciding with the initiation of frost detection by the CV method. This correlation indicates that the sensor captures the transition from condensation to frost accumulation on the HX surface. The sensor readings quickly reached a saturation point shortly after this sharp decrease, corresponding to frost accumulation as indicated by the CV method. However, beyond this saturation point, the sensor appeared to lack the ability to distinguish between moderate and severe levels of frost accumulation. This limitation suggests that while the sensor is highly effective at detecting the onset of frost, it may not provide information about the extent of frost buildup once a certain threshold is surpassed.

During the defrosting cycles, the sensor exhibited sharp decreases in resistivity, corresponding to the melting of frost and the presence of liquid water among the sensor electrodes. This response was followed by a return to the initial resistivity values once the HX surface dried. The sensor’s rapid reaction to defrosting processes shows its potential for real-time monitoring and control in refrigeration systems.

An analysis of the steady-state region before defrosting revealed a relationship between decreasing temperature and increasing sensor resistance. The calculated TCR were negative across all cycles, indicating that the resistance of the conductive polymer composite increased as the temperature decreased. This behavior is consistent with the characteristics of conductive polymer composites, where charge carrier mobility diminishes at lower temperatures due to reduced thermal energy facilitating electron movement. The observed TCR values highlight the need to consider temperature effects when interpreting sensor readings, particularly during periods of stable frost accumulation.

The current study does not include a controlled calibration to fully decouple the influence of temperature from frost on the sensor’s resistance readings. While temperature fluctuations during the tests were not negligible, their impact on frost detection is expected to be limited. This is because frost detection is primarily characterized by a sudden resistance change caused by the presence of frost, which occurs even when temperature changes are more gradual.

However, it is important to acknowledge that other factors may influence the sensor’s resistance during this period. Variations in ambient humidity can affect the moisture content within the frost layer, potentially altering its electrical properties and, consequently, the sensor’s resistance readings. Environmental conditions such as slight fluctuations in airflow or intake air temperature could also indirectly influence sensor readings by altering the rate of frost accumulation or affecting the thermal properties of the sensor and HX surface.

The high-frequency fluctuations observed in the resistance data are attributed to the limitations of the voltage divider circuit at high resistance values. As the voltage divider $V_{out}$ approaches the upper limits of the ADC resolution, small voltage changes result in significant calculated resistance variations when applying Equation (2). This phenomenon introduces noise into the resistance measurements, potentially obscuring underlying trends. The application of a moving average to the resistance data helped mitigate these fluctuations, revealing more coherent patterns and facilitating a clearer analysis of the sensor’s behavior.

Comparatively, the CV method provided smooth and consistent readings across all tests, serving as a reliable reference for frost detection. While the sensor data exhibited more complexity due to the factors discussed, observable patterns correlating with the frost accumulation and removal processes were evident. The sensor’s ability to detect frost formation concurrently with or slightly before the CV method suggests that it can serve as an effective tool for real-time frost monitoring. However, the lack of differentiation between moderate and severe frost levels beyond the saturation point indicates a need for further refinement if detailed frost accumulation profiling is required.

The analysis of ADC values and temperature data for a whole cycle revealed that a correlation between temperature and ADC readings is observable mostly during the steady-state region before defrosting. During phases of condensation, frost formation, and defrosting, no apparent relationship exists between these two variables. This indicates that while temperature variations are expected to always influence sensor readings, their influence is minimal compared to other factors that have a more significant effect. Consequently, temperature is not the predominant factor affecting ADC fluctuations during periods of rapid environmental changes. In these non-steady-state phases, factors such as the physical presence of condensation, frost accumulation, air, and water during defrosting have a more substantial impact on the sensor’s readings than temperature alone.

In practical applications, the sensor’s prompt responsiveness to frost formation and defrosting events can contribute to optimizing defrost cycles in refrigeration systems. By providing immediate feedback on the presence of frost and the effectiveness of defrosting procedures, the sensor can enable demand-based defrost control, reducing energy consumption and improving system efficiency. The sensor’s limitations in distinguishing between varying levels of heavy frost may be mitigated by integrating it with additional sensing methods or by calibrating the system to recognize specific saturation thresholds corresponding to maintenance needs.

## 5. Conclusions

In this study, a 3D-printed resistive frost-detection sensor was developed and evaluated for its effectiveness in detecting the onset of frost formation and responding to defrosting events in refrigeration systems. The sensor demonstrated consistent responsiveness across all experimental cycles, capturing the transition from condensation to frost accumulation and exhibiting rapid reactions during the defrosting processes. The use of additive manufacturing allowed for a flexible and customizable sensor design, enabling easy integration into the heat exchanger geometry without significant disruption to airflow.

While the sensor effectively detected the initial formation of frost, it reached a saturation point beyond which it could not apparently distinguish between moderate and severe levels of frost accumulation. This limitation indicates that the sensor, in its current form, is more suitable for identifying the onset of frost rather than quantifying its extent. Additionally, temperature variations were found to influence sensor readings during the steady-state region before defrosting, with the resistance of the conductive polymer composite increasing as the temperature decreased. This behavior is consistent with the characteristics of conductive polymers and underscores the importance of accounting for temperature effects in sensor calibration and data interpretation.

To enhance the sensor’s ability to quantify the extent of frost accumulation beyond initial detection, future research should focus on exploring alternative electrode patterns and dimensions. Investigating designs that optimize the interdigital electrode spacing to accommodate varying frost thicknesses and densities could reduce signal saturation and improve the sensor’s range and resolution. Additionally, calibrating the sensor to account for the effects of both temperature variations and frost properties would provide a more accurate correlation between sensor readings and frost accumulation.

Exploring the effects of environmental factors such as humidity and intake air temperature on sensor performance would provide a more comprehensive understanding of its operational context. Controlled experiments that systematically vary these parameters could elucidate their impact on resistance measurements and inform strategies to mitigate their influence. Advancements in the voltage measurement system, such as employing higher-resolution ADCs or optimizing the voltage divider circuit, could reduce measurement noise and enhance the reliability of resistance data.

Furthermore, creating a control strategy using this sensor data could optimize defrosting operations. Although the sensor does not detect high amounts of frost, a relationship could be established between the time taken for the sensor to reach frost-detection saturation and the appropriate time to initiate defrosting. Factors such as system operation duration, the interval between initial detection and sensor saturation, and the necessary defrosting time are all related to frost formation conditions and parameters influencing this process.

## 6. Patents

The patent submission process is underway.

## Data Availability

Data are available upon request.

## List of Symbols and Abbreviations

**Sym./Abbr.**	**Meaning**	**Unit**
ADC	Analog-to-Digital Converter reading	$\left[-\right]$
HX	Heat exchanger	N/A
ΔR	Resistance variation	$\left[\Omega \right]$
$R_{0}$	Reference resistance	$\left[\Omega \right]$
$R_{f}$	Fixed resistor resistance	$\left[\Omega \right]$
$R_{s}$	Sensor resistance	$\left[\Omega \right]$
RH	Relative humidity	$\left[\%\right]$
ΔT	Temperature variation	$\left[K\right]$
T	Temperature	$\left[K\right]$
TCR	Temperature Coefficient of Resistance	$\left[ppm/K\right]$
$V_{in}$	Input voltage to the voltage divider circuit	$\left[V\right]$
$V_{out}$	Output voltage from the voltage divider circuit	$\left[V\right]$
